# Individuals with and without child maltreatment experiences are evaluated similarly and do not differ in facial affect display at zero- and first-acquaintance

**DOI:** 10.1186/s40479-023-00222-3

**Published:** 2023-05-21

**Authors:** Lara-Lynn Hautle, Jennifer Kurath, Lena Jellestad, Antonia M. Lüönd, Tanja S. H. Wingenbach, Sascha Frühholz, Billy Jansson, Inga Niedtfeld, Monique C. Pfaltz

**Affiliations:** 1 Department of Consultation-Liaison Psychiatry and Psychosomatic Medicine, University Hospital Zurich, Zurich, Switzerland; 2 Medical Faculty, University of Zurich, Zurich, Switzerland; 3 School of Human Sciences, Faculty of Education, Health, and Human Sciences, University of Greenwich, London, UK; 4 Department of Psychology, Cognitive and Affective Neuroscience, University of Zurich, Zurich, Switzerland; 5 Department of Psychology and Social Work, Mid Sweden University, Östersund, Sweden; 6 Department of Psychosomatic Medicine, Medical Faculty Mannheim at, Central Institute of Mental Health, Heidelberg University, J5, 68159 Mannheim, Germany

**Keywords:** Child maltreatment, Zero-acquaintance, First-acquaintance, Facial emotion expression

## Abstract

**Background:**

Individuals with a history of child maltreatment (CM) are more often disliked, rejected and victimized compared to individuals without such experiences. However, contributing factors for these negative evaluations are so far unknown.

**Objective:**

Based on previous research on adults with borderline personality disorder (BPD), this preregistered study assessed whether negative evaluations of adults with CM experiences, in comparison to unexposed controls, are mediated by more negative and less positive facial affect display. Additionally, it was explored whether level of depression, severity of CM, social anxiety, social support, and rejection sensitivity have an influence on ratings.

**Methods:**

Forty adults with CM experiences (CM +) and 40 non-maltreated (CM-) adults were filmed for measurement of affect display and rated in likeability, trustworthiness, and cooperativeness by 100 independent raters after zero-acquaintance (no interaction) and 17 raters after first-acquaintance (short conversation).

**Results:**

The CM + and the CM- group were neither evaluated significantly different, nor showed significant differences in affect display. Contrasting previous research, higher levels of BPD symptoms predicted higher likeability ratings (*p* = .046), while complex post-traumatic stress disorder symptoms had no influence on ratings.

**Conclusions:**

The non-significant effects could be attributed to an insufficient number of participants, as our sample size allowed us to detect effects with medium effect sizes (f^2^ = .16 for evaluation; f^2^ = .17 for affect display) with a power of .95. Moreover, aspects such as the presence of mental disorders (e.g., BPD or post-traumatic stress disorder), might have a stronger impact than CM per se. Future research should thus further explore conditions (e.g., presence of specific mental disorders) under which individuals with CM are affected by negative evaluations as well as factors that contribute to negative evaluations and problems in social relationships.

## Background

Child maltreatment (CM) is a global concern that has been linked to severe mental health problems [1]. CM is defined as abuse and neglect that occurs to children under the age of 18. It includes all types of physical and/or emotional ill-treatment, sexual abuse, neglect, negligence and commercial or other types of exploitation, resulting in actual or potential harm to the child’s health, survival, development, or dignity in the context of a relationship of responsibility, trust or power [2]. Individuals with a history of CM are at increased risk to develop behavioural, physical, and mental health problems [2–5]. Furthermore, studies demonstrated that children and adults affected by CM suffer from a broad range of social problems [6, 7]. For example, individuals with CM are more often disliked, rejected and victimized by their peers and teachers compared to individuals without CM experiences [8–11]. Similarly, patients with borderline personality disorder (BPD), a population with a high prevalence of CM experiences [12], have previously been evaluated as less trustworthy, less likeable, and less cooperative compared to healthy controls by raters who were left blind to their disorder [13]. Such negative evaluations and experiences of peer rejection likely reinforce poor relationship satisfaction, which is common in individuals with CM (e.g., [14–16]). This is alarming, given that close relationships can protect from negative consequences of stress and increase well-being [17]. To support survivors of CM in establishing and maintaining close, healthy and satisfying relationships, it is important to identify the factors underlying negative evaluations through others.

One aspect that may add to the abovementioned negative evaluations is emotion expression. The facial expression of emotions, as a key component of communication in social interaction [18], is stimulated by interactions with significant others during childhood [19]. Consequently, emotionally unavailable or abusive primary caregivers may alter the development of emotion expression, as has been shown in a previous study where women with sexual abuse experiences expressed fewer emotions in the face while watching emotion-eliciting film stimuli [20]. Expressing facial emotions is not only essential for the communication of emotions but also for social connectedness [21]. Generally, the tendency to approach and interact with someone displaying a positive facial expression is higher compared to when a negative emotion is expressed [22, 23]. Facial emotion expression might thus affect how one is perceived by others, which in turn may also account for difficulties in establishing close and satisfying relationships.

To date, studies investigating alterations in facial emotion expression in individuals with a history of CM are scarce. However, populations with a high prevalence of CM (such as post-traumatic stress disorder (PTSD) [24] or BPD; [12]) have been found to show alterations in emotion expression. For example, in a study by Kirsch and Brunnhuber (2007) [25], PTSD patients displayed more expressions of anger during a psychodynamic interview while a healthy control group more frequently displayed happy facial expressions. Similarly, Hepp, Storkel, Kieslich, Schmahl, and Niedtfeld (2018) videotaped individuals with and without BPD while answering questions about personal preferences. The authors found that individuals with BPD were rated to display significantly more negative and significantly less positive affect in comparison to those without BPD. In a second study, the authors showed that negative evaluations of individuals with BPD were mediated by less positive and more negative facial emotional display [26].

Alterations in facial emotion expression in individuals with a history of CM are to date poorly understood. Thus, the goal of the current study was to examine whether possible negative evaluations of adults with CM carried out by independent raters naïve to their trauma history at zero- (without interaction) and first- (after a short interaction) acquaintance would be mediated by altered facial emotion expressions. Based on the results of Hepp and colleagues (2019), we selected negative affect (NA; sad, angry, scared, disgusted) and positive affect (PA; happy) display as potential mediators in the relationship between CM and ratings of likeability, trustworthiness, and cooperativeness. Specifically, and in line with prior research [26], we hypothesized that adults with a history of CM would facially express more NA and less PA compared to individuals without CM experiences and that these differences in NA and PA display would mediate the association between CM experiences and negative evaluations on the traits likeability, trustworthiness, and cooperativeness. In line with findings by Hepp and colleagues (2018), we expected individuals with CM experiences not to differ in objective cooperativeness, measured with an economic game [27]. In exploratory analyses, we explored whether the level of depression, severity of CM, social anxiety, social support, and rejection sensitivity have a negative impact on ratings, in addition to CM and facial expression. We hypothesized that higher levels on each scale would negatively influence likeability, trustworthiness, as well as cooperativeness. The aims, hypotheses, design, and analyses for this study were pre-registered at *aspredicted.org* prior to data collection under the title “Negative Evaluation of individuals with a history of child maltreatment” (#83,676). The pdf is available from https://aspredicted.org/b7mn5.pdf.

## Materials and methods

The study was approved by the local ethics committee (Kantonale Ethikkomission Zürich) and conducted as part of an overarching project on socio-emotional consequences of CM. The study was conducted in two steps: 1) creation of stimulus material (video recordings of target participants with and without a history of CM) and evaluation by confederates (members of the study team) during a first-acquaintance paradigm (FAP), involving an interaction between target participants and confederates; 2) evaluation of video recordings by independent raters (zero-acquaintance paradigm), involving no interaction between target participants and raters. All participants from both step 1 and step 2 gave written informed consent prior to participation.

### Participants

Participants of step 1 (individuals with and without self-reported history of CM = target participants) were recruited via online social media platforms, flyers, mailing lists, from a study pool, and in collaboration with out-patient clinics in the area of Zurich. Individuals aged 18–65 years with normal or corrected-to-normal vision that are native German speakers (or equivalent) were included. Individuals were assigned to the CM + group (participants with CM experiences) based on the Childhood Trauma Questionnaire short form (CTQ-SF; [28] for further description see Psychometric Assessment section). For this study, individuals were classified into the CM + group when meeting the cut-off values of “none / minimal” in at least one of the subscales according to Bernstein and colleagues (2003). Individuals scoring below these cut-offs in all subscales were assigned to the CM- group (participants without CM experiences). Exclusion criteria were antipsychotic, benzodiazepine, or tricyclic antidepressant medication, acute suicidality, lifetime psychotic symptoms, substance abuse or dependency (past 12 months), pregnancy, and physical health problems affecting psychophysiological measurements (these measurements were conducted as part of the overarching project). The initial sample of step 1 consisted of almost twice as many target participants in the CM + group (*n* = 70) than the CM- group (*n* = 40), possibly due to the specific mentioning of “child maltreatment experiences” in the recruitment announcements. To match the two groups (i.e., to enable comparability between groups regarding gender, age, and education level), a random selection by matched subgroups was applied, resulting in a final sample of 40 target participants (26 female) in the CM + and 40 target participants (25 female) in the CM- group. Target participant’s characteristics are visualised in Table 1.

**Table 1:** Summary statistics of the Target participants’ characteristics

	**CM + **(*n* = 40)		**CM-** (*n* = 40)		
	***n***	***%***	***n***	***%***	**Group comparison**
Female gender	26	65.0	25	62.5	ns
Anxiety disorders^a^	10	25.0	4	10.0	ns
Obsessive–compulsive disorder^a^	4	10.0	1	2.5	ns
Affective disorders^a^	6	15.0	1	2.5	ns
Eating disorders^a^	0	0.0	1	2.5	ns
Sleeping disorders^a^	6	15.0	0	0.0	χ^2^ [1] = 6.49 ^*^
SSRI medication	6	15.0	1	2.5	ns
Other antidepressant medication	4	10.0	0	0.0	ns
	***M***	***SD***	***M***	***SD***	
Age (years)	33.00	13.69	32.85	12.46	ns
Educational group	2.46	0.82	2.4	0.78	ns
BDI-2	10.75	9.43	3.45	3.62	CM + > CM-; *U* = 353.0 ^***^
MSI-BDI	3.98	2.59	0.88	1.34	CM + > CM-; *U* = 260.0 ^***^
CTQ emotional neglect	16.37	5.11	6.53	1.39	CM + > CM-; *U* = 060.5 ^***^
CTQ physical neglect	8.35	3.06	5.18	0.39	CM + > CM-;* U* = 225.5 ^***^
CTQ emotional abuse	12.00	5.34	5.50	0.85	CM + > CM-;* U* = 164.0 ^***^
CTQ physical abuse	8.13	4.28	5.13	0.34	CM + > CM-;* U* = 435.0 ^***^
CTQ sexual abuse	8.53	6.13	5.00	0.00	CM + > CM-;* U* = 460.0 ^***^
PDS	2.28	2.11	0.98	1.27	CM + > CM-; *U* = 680.0 ^***^

For count data comparison chi-square test and Fisher's exact test were used. Educational groups consisted of 4 levels: 1 = 11 years of education, 2 = 14–15 years of education, 3 = 19–22 years of education and 4 = 24–26 years of education. All p-values were computed two-sided. *CM* + Child maltreatment group, *CM-* Non child maltreatment group, *ns* Non-significant, * = *p* < .05, *** = p < .001, ^a^ assessed with Mini-DIPS, *MSI-BPD* Dimensional Borderline Personality Disorder score, *SSRI* Selective Serotonin Reuptake Inhibitor, *BDI-2* Beck Depression Inventory 2, *CTQ* Childhood Trauma Questionnaire, *PDS* Post-Traumatic Stress Diagnostic Scale

The final sample of the confederates of step 1 consisted of 17 psychology student raters (11 female), with a mean age of 25.9 years, and a mean education level of 2.22 (representing the Swiss schooling system, see further description in Psychometric Assessment section).

Participants (raters) of step 2 of the study were recruited via online social media platforms, mailing lists, and from a pool of former study participants. Individuals aged 18–65 years with normal or corrected-to-normal vision that are native German speakers (or equivalent) were included. The final sample consisted of 100 raters (67 female). Raters had a mean age of 28.8 years and a mean education level of 2.53 (representing the Swiss schooling system, see further description in Psychometric Assessment section)

### Psychometric assessment

CM was measured with the *German version of the Childhood Trauma Questionnaire, short form* (*CTQ-SF*; 28), German translation and validation of Bader et al., 2009 [29] to categorize target participants into CM + and CM- group in step 1. Internal consistency for the subscores is high (α > 0.81), except for the physical neglect subscale (α = 0.49). Nevertheless, the CTQ-SF is a widely used measurement [30].

During step 1, the target sample underwent the following additional assessments: 1) Depressive symptoms were measured using the German version of the *Beck’s Depression Inventory 2* (*BDI-II*; [31]). It is a self-report measure for the assessment of the severity of depressive symptoms over the past week and comprises 21 items, which can be added up to a sum score of 0–63, with a good validity and reliability [32]. 2) Current mental disorders (affective disorders, obsessive–compulsive disorders, anxiety disorders, eating disorders, sleeping disorders) were assessed using the diagnostic interview for mental disorders *Mini Diagnostisches Interview bei psychischen Störungen* (*Mini-DIPS*; [33]). The Mini-DIPS is a short, semi-structured clinical interview to assess the most common mental disorders (excluding personality disorders) according to the DSM-5. 3) The number of experienced trauma types was assessed using the trauma checklist of the *Posttraumatic Diagnostic Scale* (*PDS*; [34]). This section of the instrument corresponds to stressor criterion A of the DSM-5 for PTSD and demonstrates excellent internal consistency and test–retest reliability, and good convergent validity with the PTSD Checklist—Specific Version and the PTSD Symptom Scale—Interview Version for DSM–5 [35]. If a participant had one or more traumatic experience, they completed the *International Trauma Questionnaire – German Version* (*ITQ*; [36]). The ITQ is a short questionnaire aiming to assess PTSD and complex PTSD symptoms following simple diagnostic rules [36]. 4) For the assessment of BPD, the *McLean screening instrument for borderline personality disorder* (*MSI-BPD*; [37, 38]) was used. This self-report measure is a screening instrument based on a subset of the questions that comprise the borderline module of the Diagnostic Interview for DSM-IV personality disorders, yielding both good sensitivity and specificity for the diagnosis of DSM-IV BPD [37]. 5) Social interaction anxiety was measured with the *Social Interaction Anxiety Scale* (*SIAS*; [39]), a self-report questionnaire assessing social interaction anxiety defined as “distress when meeting and talking with other people” and includes 20 items on a 5-point Likertscale. It shows good reliability (retest-reliability: > 0.90; Cronbach’s alpha = 0.86) [39]. 6) Social support was measured using the *Fragebogen zur sozialen Unterstützung* (*F-SozU K22*; [40]). This self-report questionnaire assesses social support with 22 items and shows good reliability (Cronbach’s alpha = 0.81-0.93) [40]. 7) Rejection sensitivity was assessed with the *Rejection Sensitivity Questionnaire* (*RSQ*; [41, 42]), which is a self-report questionnaire assessing trait rejection sensitivity with 18 items. It shows good reliability and validity [41].

Additionally, all participants’ educational levels were evaluated. Four categories were used; 1 = up to 13 years of education (mandatory school years), 2 = up to 18 years of education (high school degree), 3 = up to 23 years of education (university degree; Bachelor or higher) and 4 = more than 23 years of education (university degree; PhD or higher).

### Material

#### Production of stimulus material and zero-acquaintance (thin slices) paradigm

The stimulus material comprised videos of 40 target participants of the CM + group and 40 target participants of the CM- group. All target participants performed the thin slices paradigm (TSP) [13, 43] while being filmed. In this paradigm, target participants were asked about their favourite meal, colour, hobby, book, movie, animal, past vacation, and holiday destination, while sitting in front of a white wall. Targets could freely decide whether they wanted to just name their answer to each category or provide further explanation. After the videos had been collected, sound and video track were separated from each other, and videos were cut at 30 s. In part 2, videos were presented to the independent raters without audio trace to exclude potential effects of speech content or prosody, based on the procedure by Hepp and colleagues (2018).

#### First-acquaintance paradigm

During the FAP, target participants held a short three-minute conversation with a same sex confederate via skype for business. The online interaction (rather than an in-person interaction) was chosen due to regulatory aspects of the Covid-19 pandemic (mandatory use of facemasks, which might have critically hampered the interpretation of facial emotion expression). The three-minute interaction consisted of a standardized small-talk conversation. Target participants were told that the interaction partner was another study participant in order to create a close to real-life condition. Confederates had a set of questions and answers (e.g., “have you participated in a study before?”, “yes, this is my second participation”, “do you live in Zurich?” etc.) which they went through sequentially. If all questions had been asked, confederates initiated no more conversation. After three minutes, the experimenter broke off the dialogue. Directly after the interaction, target participants were debriefed.

#### Trustworthiness, likeability, and cooperativeness ratings

Raters of step 2 watched all 80 target videos (presented electronically using E-Prime 3.0 software (Psychology Software Tools, Pittsburgh, PA) [44]) and rated targets on likeability, trustworthiness, and cooperativeness on a 7-point Likert scale. Similarity ratings were also collected on a 7-point Likert scale. After rating 40 of the videos, there was a 10-min break in which the participants were allowed to step outside and walk around but were asked to refrain from using their mobile phone in order to prevent any exposure to potential emotional content. Confederates of the FAP rated the target participants identically on likeability, trustworthiness, cooperativeness, as well as similarity on a 7-point Likert scale.

To measure the target participant’s objective cooperativeness, they took part in the *dictator game* (DG; 27). The dictator game is an economic game to assess cooperative behaviour. A fixed amount of money (here: 20 Swiss Francs in 1 Franc coins) has to be divided between oneself and an unknown third person. Participants distribute the money in private and are informed that someone unknown to them (i.e., not the person who serves as their experimenter) will open the envelope at the end of the participation. They are furthermore notified that the allocated amount will remain anonymous to both the experimenter and the recipient.

#### FaceReader™

Objective measure of PA and NA display was assessed with the software FaceReader™ version 8 [45]. To determine the overall intensity of each emotion detected, FaceReader™ provided us with a “detailed log” where, with a continuous scale measure, the intensity of different emotions at every given time are recorded. The mean% (average intensity) of each emotion over the 30 s period was then calculated. Each video was calibrated manually, and the sample rate was set to every second frame as suggested by the FaceReader™ manual 8 [46]. The FaceReader™ is a valid measurement tool for emotional facial expressions, with 88% accuracy [45].

### Procedure

The overarching project comprised two laboratory appointments. First, eligible targets were screened via telephone for inclusion and exclusion criteria and then scheduled an appointment for a first assessment in the laboratory, during which graduate psychology students trained and supervised by an experienced licensed psychotherapist (last author), assigned the questionnaires (SIAS, F-SozU K22, RSQ, BDI-II, MSI-BPD, ITQ) and conducted clinical interviews (CTQ-SF, Mini-Dips, PDS checklist). Target participants received a written study information and signed an informed consent form. The second laboratory visit comprised several emotion recognition paradigms (part of the overarching project not assessed for the current study), a personal space paradigm (not assessed for the current study; for further description see Hautle et al. (under review), as well as the above described TSP, DG, and FAP conducted for part 1 of the current study. At the end of the second visit, we debriefed participants. They were reimbursed with 20 Swiss Francs per hour for their participation in each study visit.

For part 2, eligible raters scheduled an appointment for the assessment in our laboratory. Each rater completed an informed consent form and then rated all 80 target videos, collected in step 1. At the end of the visit, participants received either course credits (1 credit per hour) or monetary compensation (20 Swiss Francs per hour) for their participation.

### Planned statistical analyses

Statistical analyses were calculated in R, version 4.2.1 (R Core Team, 20,122). As pre-registered, it was planned to add similarity ratings to all models as a control variable. The first set of models to test Hypothesis 1 (individuals with CM experiences are evaluated as less likeable, trustworthy, and cooperative by independent raters at zero-acquaintance, compared to unexposed controls) comprised three separate regression analyses via lm function using the stats package for the influence of group allocation (CM + vs. CM-; predictor variable) on each criterion variable, i.e. average likeability, trustworthiness, and cooperativeness ratings by independent raters from part 2. The second set of models were related to Hypothesis 2 (individuals with CM experiences are evaluated as less likeable, trustworthy, and cooperative by confederates at first-acquaintance, compared to unexposed controls), planned to be tested by three separate regression analyses, with the ratings by confederates as criterion variables. As confederate ratings were missing for four participants (two of the CM- and two of the CM + group), a total of 76 ratings were collected. Attractiveness ratings were planned to be additionally added to models of confederate ratings as a control variable.

To test Hypothesis 3 (individuals with CM experiences express less PA and more NA compared to unexposed controls), five separate regression analyses via lm function for the influence of group (predictor variable) on each emotion display (criterion variables; happy, sad, angry, scared, disgusted) were intended to be conducted.

For exploratory analyses, a t-test with independent samples was conducted to test whether targets differed in their objective cooperativeness (as assessed with the DG). Furthermore, fifteen regression analyses were conducted to test whether the three rating dimensions (criterion variables) were related to self-reported levels of depressive symptoms, severity of CM, social anxiety, social support, and rejection sensitivity (all dimensional predictors).

Finally, the planned (according to pre-registration) mediation models between ratings and group, as well as overall levels of emotion expression, were not conducted, as no significant differences between study groups in ratings or affect display were found (see section Results). Instead, exploratory (non-preregistered) analyses were conducted to better understand the unexpected findings and their deviation from previous research [8, 25, 26]. More specifically, a possible influence of BPD (dimensional predictor) and complex PTSD symptoms (dimensional predictor) on each of the three rating dimensions (criterion variables), were assessed via lm function.

## Results

Univariate analyses used to explore the relationships between the main variables of zero- and first-acquaintance ratings, as well as for emotion display revealed that there was no significant effect of group on our dependent variables (see Table 2). Thus, further multivariate regression analyses that involve the inclusion of the covariates (similarity and attractiveness) were not warranted (and thereby deviating from our analysis plan). For objective measurement of emotion display, FaceReader™ analyses accurately detected emotional facial expressions, with a total of only 5% not recognized expressions, as labelled “unknown” by FaceReader™.

**Table 2:** Descriptive Statistics, *p*-values (and Cohen’s *d*) for Zero-, First-Acquaintance and Affect

**Variables**	**CM + **(*n* = 40)		**CM-** (*n* = 40)		**Group comparison**
	***M***	***SD***	***M***	***SD***	***p (d)***
**Zero-acquaintance**					
Likeability	4.37	0.64	4.15	0.67	.14 (.34)
Trustworthiness	4.62	0.51	4.49	0.56	.28 (.24)
Cooperativeness	4.59	0.58	4.46	0.55	.31 (.23)
Similarity	2.92	0.51	2.81	0.57	.37 (.20)
**First-acquaintance**					
Likeability	5.08	1.21	5.46	1.14	.15 (-.32)
Trustworthiness	5.32	1.06	5.56	0.97	.29 (-.24)
Cooperativeness	5.32	0.97	5.51	0.94	.38 (-.19)
Similarity	4.51	1.26	4.38	1.55	.68 (.09)
Attractiveness	4.51	1.37	4.51	1.50	.99 (.00)
**Emotion display**					
Happy^a^	11.55	0.13	9.00	0.09	.48 (2.36)
Sad^a^	1.56	0.02	2.10	0.03	.11 (.00)
Angry^a^	1.37	0.03	2.00	0.03	.36 (-.33)
Scared^a^	0.91	0.01	1.55	0.02	.32 (-.50)
Disgusted^a^	1.03	0.02	0.51	0.01	.54 (.00)

All *p*-values were computed two-sided. ^a^ average intensity (in %) per clip

### Secondary and Exploratory Analyses

The t-test with independent samples to test whether targets differed in their objective cooperativeness was not significant (*p*_*two-tailed*_ = 0.45), indicating that the CM + and the CM- group did not significantly differ in the amount of money they shared with an unknown person during the DG.

None of the pre-registered exploratory analyses revealed significant results, see Table 3 for a summary. Similarly, none of the additional exploratory analyses (not pre-registered) were significant, apart from the influence of BPD on likeability in the zero-acquaintance paradigm with the predictor explaining 5% of the variance (*R*^2^ = 0.050, *F*(1, 78) = 4.13, *p* = 0.046). Unexpectedly, more BPD symptoms were associated with higher likeability ratings (*p* = 0.046, β =  + 0.06). Furthermore, as results were unexpected, separate sensitivity analyses for a power of 0.95, 0.90, and 0.80, using a linear multiple regression, R^2^ increase, for ratings and R^2^ deviation from zero for affect display, using the G*Power tool [47] were conducted post-hoc.

**Table 3:** Exploratory Analyses

		**Estimated (se)**			
	**Predictor**	**Intercept**	**Unstandardized β**	***t***	***p***
Likeability	BDI-2	4.23 (0.09)	0.00 (0.00)	.47	.64
	CTQ	4.12 (0.19)	0.00 (0.00)	.79	.43
	SIAS	4.21 (0.16)	0.00 (0.00)	.30	.76
	SOZU	2.95 (1.25)	0.37 (0.35)	1.05	.29
	RSQ	4.31 (0.19)	-0.01 (0.04)	-.27	.79
Trustworthiness	BDI-2	4.56 (0.08)	-0.00 (0.00)	-.16	.88
	CTQ	4.48 (0.15)	0.00 (0.00)	.51	.61
	SIAS	4.57 (0.13)	-0.00 (0.00)	-.16	.87
	SOZU	3.42 (1.01)	0.32 (0.28)	1.13	.26
	RSQ	4.67 ( 0.16)	-0.03 (0.03)	-.77	.44
Cooperativeness	BDI-2	4.53 (0.09)	-0.00 (0.01)	-.12	.90
	CTQ	4.46 (0.16)	0.00 (0.00)	.43	.67
	SIAS	4.53 (0.14)	-0.00 (0.00)	-.02	.99
	SOZU	3.42 (1.06)	0.31 (0.29)	1.04	.30
	RSQ	4.59 (0.17)	-0.01 (0.03)	-.44	.66

*BDI-2* Beck Depression Inventory 2, *CTQ* Childhood Trauma Questionnaire dimensional score, *SIAS* Social Interaction Anxiety Scale, *SOZU* F-SozU K22, Fragebogen zur sozialen Unterstützung (social support questionnaire), *RSQ* Rejection Sensitivity Questionnaire

Sensitivity power analyses for the ratings showed that our sample size allowed us to detect effect sizes of f^2^ = 0.16 with a power of 0.95, an effect size of f^2^ = 0.13 with a power of 0.90, and an effect size of f^2^ = 0.09 with a power of 0.80. For affect display, sensitivity power analyses showed that our sample size allowed us to detect effect sizes of f^2^ = 0.17 with a power of 0.95, an effect size of f^2^ = 0.13 with a power of 0.90, and an effect size of f^2^ = 0.10 with a power of 0.80.

## Discussion

This study aimed to assess whether more negative evaluations of individuals with CM experiences compared to unexposed individuals would be mediated by less positive and more negative affect display in a zero- and first acquaintance paradigm. Unexpectedly, none of our hypotheses were confirmed. No strong evidence was detected for differences in evaluation, nor for differences in affect display for none of the emotions (happy, sad, angry, scared, disgusted) between the CM + and the CM - group. Additional exploratory analyses revealed that higher BPD symptoms were correlated with higher scores in likeability solely at zero-acquaintance.

### Conclusion

This study found no difference of evaluation between adults with and without CM as well as no mediating effect of affect displays. Possibly, other aspects such as the presence of mental disorders (e.g., BPD or PTSD [13, 25, 26]), have a stronger impact on negative evaluations than CM per se. Indeed, recent studies showed that CM combined with mental disorders (e.g., depression, social anxiety) have an influence on socially relevant functions such as e.g., emotion recognition and the regulation of closeness and distance (Hautle et al., [48], in press; Hautle et al., under review). Perhaps, similar processes are at play when it comes to emotion expression. It would be important for future research to investigate the combination of CM with specific diagnoses like complex PTSD, depression or BPD (rather than measuring mental disorder symptoms likely leading to subclinical samples as done in this study) and their influence on facial affect display and evaluation. Such studies could contribute to better understand the conditions under which negative evaluations of individuals with CM occur and might identify possible contributors to negative evaluations of those affected by CM. On the long run, such research might help to counteract experiences of rejection and victimization, foster positive and satisfying relationships and thereby increase mental and physical well-being.

### Ratings

Post-hoc sensitivity power analyses for ratings revealed that at least medium effects could be detected with the given sample of raters in the study, and a substantially larger sample size would be needed in order to detect small effects. Although analyses of group differences were not significant, descriptively, the CM + group displayed more positive and less negative (sad, angry, and scared) affect, and was rated higher in likeability and trustworthiness, than the CM - group at zero-acquaintance. Hepp and colleagues (2019) demonstrated that affect display is linked to how individuals are perceived by others. However, in contrast to the current study, Hepp and colleagues’ (2019) results were statistically significant. Individuals with BPD were rated as showing less PA and more NA, and PA mediated the association between BPD and likeability as well as trustworthiness, while NA mediated the association between BPD and trustworthiness [26].

Interestingly, results regarding first-acquaintance differed somewhat from results regarding zero-acquaintance in the current study. Descriptively, the CM + group was rated lower in likeability, trustworthiness, and cooperativeness by confederates. Video analyses for affect display were conducted using videos from the TSP only and general affect display in the two different paradigms (first- and zero-acquaintance) may not have coincided. Thus, it might be possible that the CM + group expressed more negative and less positive affect during the FAP but not during the TSP, which might have led to a (non-significant) less positive evaluation during the FAP by the confederates.

Furthermore, results could be explained by methodological shortcomings. Videos shown to raters resulted in a rather long-lasting evaluation procedure, even though each video was only 30 s long. The whole paradigm approximately lasted one hour (including the break). It is likely that raters started to feel bored, since it was a relatively monotonous task [49]. As boredom has been proposed to be an unpleasant affective state [49], it might have impacted the ratings of target participants. This would also be in line with general rating differences found between confederate raters from the FAP and independent raters from the zero-acquaintance paradigm.

Contrasting previous studies [13], our exploratory analysis revealed that individuals with higher BPD scores were rated as significantly more likeable as individuals with lower BPD scores independent of group allocation. This result was very surprising as individuals with BPD have previously been found to be evaluated as less likeable, less cooperative, and less trustworthy in comparison to healthy controls in the TSP [13]. However, BPD symptom scores in our sample were rather low, given that 8 out of 10 was the highest score and was only reached by three participants (of the CM + group). In contrast, participants from an inpatient and outpatient unit in the study by Hepp and colleagues (2018) demonstrated a symptom severity similar to patient samples in other studies (see [50]), which is clearly higher in comparison to our (non-BPD specific) sample. Furthermore, we used a different measure for BPD than Hepp and colleagues (2018). Even though the MSI-BPD has both good sensitivity and specificity for the diagnosis of DSM-IV BPD [38], it might not have depicted the full range of BPD symptoms. Rather than indicating BPD symptoms on a Likert scale, as for example done in the Borderline Symptom List- 23 [50] used in the study by Hepp and colleagues (2018), participants in the current study rated each item in the MSI as “present” or “absent”, which may not adequately reflect the (dimensional) nature of BPD symptoms and thus might have impacted on the results of our exploratory analysis.

### Facial affect display

As for affect display, post-hoc sensitivity power analyses revealed that at least medium effects could be detected with the given sample of target participants in this study, and a substantially larger sample size is needed in order to detect small effects. Another possible reason for non-significant findings in affect display between the two groups is that the CM - group might have been more daring in showing negative or neutral facial expressions than the CM + group, who might have suppressed their negative facial expressions. As it has been shown that emotion expressions can be intentionally manipulated through learning experiences [51], it seems likely that individuals with experiences of CM have learnt to adapt to their adverse environment to protect themselves and respond adequately when interacting with their abusive or neglecting caregivers. This notion is supported by several studies. For instance, a meta-analysis by Gruhn and Compas (2020) [52] revealed that maltreatment is positively associated with emotional suppression as an emotional regulation strategy to cope with stress. One reason for this emotional strategy might lie in the fact that maltreated children expect less emotional support and practical assistance from their parents and peers in response to their emotional display, especially in the case of sadness and anger [53–55]. Though initially an adaptive strategy when growing up in a hostile family environment, suppressing one’s own emotions may not only be detrimental to future social interactions in normal environments but is also known to be predictive of higher levels of psychopathological symptoms [56]. Indeed, intentional withholding of emotional responses was found to also be a relevant dimension in other traumatized populations such as individuals suffering from PTSD [57].

When it comes to the expression of positive affect, we cannot conclusively say if emotion display was genuine or potentially masked, as we did not measure Duchenne display [58]. In the non-Duchenne smile, the eye muscle movement is lacking and is thus often called a non-enjoyment, false, fake, or social smile [59, 60]. It is believed that non-Duchenne smiles are under far more volitional control than Duchenne smiles [59, 60]. Considering that individuals with CM experience might be experienced in suppressing their feelings [52], they may also have learned to mask their emotions with expression of positive affect when actually experiencing negative affect. Indeed, it has previously been shown that non-Duchenne smile might explain the function of smiling in situations in which the expresser is actually experiencing negative affect, as when showing or masking feelings of discomfort, disliking, disappointment, embarrassment, or anxiety [61–64]. Beneficial or socially expected behaviour can be realised through deliberate expressions that can be incongruent with the actual experienced emotional state [65]. Emotions can be intensified or dampened, neutralised, or masked, depending on the context [66, 67]. During the TSP, individuals were sitting in front of a camera, knowing that they were being filmed, which might have caused more pronounced feelings such as anxiety and embarrassment in the CM + group. At the same time, individuals with CM might have successfully covered these feelings.

Moreover, our non-significant findings might be explained by differences in study samples and methods. One of the exclusion criteria in our current study was the use of tricyclic antidepressants. In the study by Hepp and colleagues (2018) over 80% of the target participants were using some form of antidepressants, which is representative for individuals with BPD, considering the high prevalence of major depressive disorder in BPD (e.g., lifetime diagnosis of 90%; e.g., [68]). However, it has previously been shown that antidepressants can lead to emotional blunting [69]. Thus, individuals of the CM + group might have experienced less emotional blunting, as only a small amount of study participants (*n* = 10) used anti-depressive medication, and hence might have expressed less negative or neutral affect. Furthermore, facial expression was measured using an objective measurement tool (FaceReader™), while Hepp and colleagues (2019) assessed PA and NA through raters. Since subjective assessments of emotion expressions seem not to match with objective assessments [70], the setting of the current study might not be comparable to previous research, where differences in affect display between clinical and control samples have been found [25, 26].

### Limitations

The study is limited by the small sample size as shown by post-hoc sensitivity power analyses. Another limitation is the retrospective self-report measurement of CM, given that is has been suggested that prospective and retrospective measures of CM identify different groups of individuals [71]. Furthermore, in line with other studies [26], we did not evaluate Duchenne display [58]. Therefore, we cannot conclusively say whether the positive affect display measured by the FaceReader™ was always genuine. Future studies should thus aim to include Duchenne display in their analyses to account for genuine positive affect display. Moreover, we used videos from the TSP for zero-acquaintance ratings, while ratings for first acquaintance were conducted during the FAP. Upcoming studies should use video material and ratings from the same paradigm to account for comparability. Our CM + group also mainly comprised participants of a community sample, with lower scores of exposure to CM compared to previous studies (e.g., [72–74]), potentially contributing to the absence of group differences. Thus, future studies might profit from a dimensional analyses approach (using CTQ-SF severity score) rather than conducting group analyses.

## Data Availability

The datasets used and/or analysed during the current study are available from the corresponding author on reasonable request.

## Abbreviations

CM: Child Maltreatment
BPD: Borderline Personality Disorder
PTSD: Post-Traumatic Stress Disorder
NA: Negative Affect
PA: Positive Affect
FAP: First-Acquaintance Paradigm
CTQ-SF: Childhood Trauma Questionnaire Short-Form
BDI-II: Beck’s Depression Inventory 2
Mini-DIPS: Mini Diagnostisches Interview bei psychischen Störungen
PDS: Post-Traumatic Stress Diagnostic Scale
ITQ: International Trauma Questionnaire
MSI-BPD: McLean Screening Instrument for Borderline Personality Disorder
SIAS: Social Interaction Anxiety Scale
F-SozU K22: Fragebogen zur sozialen Unterstützung
RSQ: Rejection Sensitivity Questionnaire
TSP: Thin Slices Paradigm
DG: Dictator Game
